# Advantages and Limitations of Current Techniques for Analyzing the Biodistribution of Nanoparticles

**DOI:** 10.3389/fphar.2018.00802

**Published:** 2018-08-14

**Authors:** Lauren Arms, Doug W. Smith, Jamie Flynn, William Palmer, Antony Martin, Ameha Woldu, Susan Hua

**Affiliations:** ^1^School of Biomedical Sciences and Pharmacy, University of Newcastle, Callaghan, NSW, Australia; ^2^Hunter Medical Research Institute, New Lambton Heights, NSW, Australia; ^3^School of Environmental and Life Sciences, University of Newcastle, Callaghan, NSW, Australia

**Keywords:** nanoparticles, nanomedicine, biodistribution, *in vivo*, imaging, techniques, advantages, limitations

## Abstract

Nanomedicines are typically submicrometer-sized carrier materials (nanoparticles) encapsulating therapeutic and/or imaging compounds that are used for the prevention, diagnosis and treatment of diseases. They are increasingly being used to overcome biological barriers in the body to improve the way we deliver compounds to specific tissues and organs. Nanomedicine technology aims to improve the balance between the efficacy and the toxicity of therapeutic compounds. Nanoparticles, one of the key technologies of nanomedicine, can exhibit a combination of physical, chemical and biological characteristics that determine their *in vivo* behavior. A key component in the translational assessment of nanomedicines is determining the biodistribution of the nanoparticles following *in vivo* administration in animals and humans. There are a range of techniques available for evaluating nanoparticle biodistribution, including histology, electron microscopy, liquid scintillation counting (LSC), indirectly measuring drug concentrations, *in vivo* optical imaging, computed tomography (CT), magnetic resonance imaging (MRI), and nuclear medicine imaging. Each technique has its own advantages and limitations, as well as capabilities for assessing real-time, whole-organ and cellular accumulation. This review will address the principles and methodology of each technique and their advantages and limitations for evaluating *in vivo* biodistribution of nanoparticles.

## Introduction

Nanomedicine is the application of nanotechnology for the diagnosis, prevention and treatment of diseases. Nanomedicines are submicrometer-sized carrier materials (nanoparticles) designed to improve the biodistribution of encapsulated compounds by delivering them more effectively and more selectively to the pathological site (site-specific drug delivery) and/or by guiding them away from potentially endangered healthy tissues (site-avoidance drug delivery) (Lammers et al., 2012). This technology aims to improve the balance between the efficacy and the toxicity of therapeutic compounds (Lammers et al., 2012). Nanoparticles can exhibit a combination of physical (e.g., size, shape, lamellarity and homogeneity), chemical (e.g., composition, surface charge, surface coating and phase transition temperature), and biological (e.g., encapsulated compounds and conjugated surface ligands) characteristics that determine their *in vivo* behavior (Bharali and Mousa, 2010; Robson et al., 2018).

Despite the significant advances in drug delivery technologies and platforms in the last several decades, the clinical translation of nanomedicines has progressed incrementally (Sercombe et al., 2015; Hare et al., 2017). It has been suggested that effective nanomedicine development requires a disease-driven approach, rather than the traditional formulation-driven approach where drug delivery system engineering has been the priority (Hare et al., 2017). This requires a strong understanding of the relationships between biology and technology, including the influence of disease pathophysiology on nanomedicine accumulation, distribution, retention and efficacy, and the correlation between delivery system properties and *in vivo* behavior in animals vs. humans (Hare et al., 2017).

**Figure 1:**
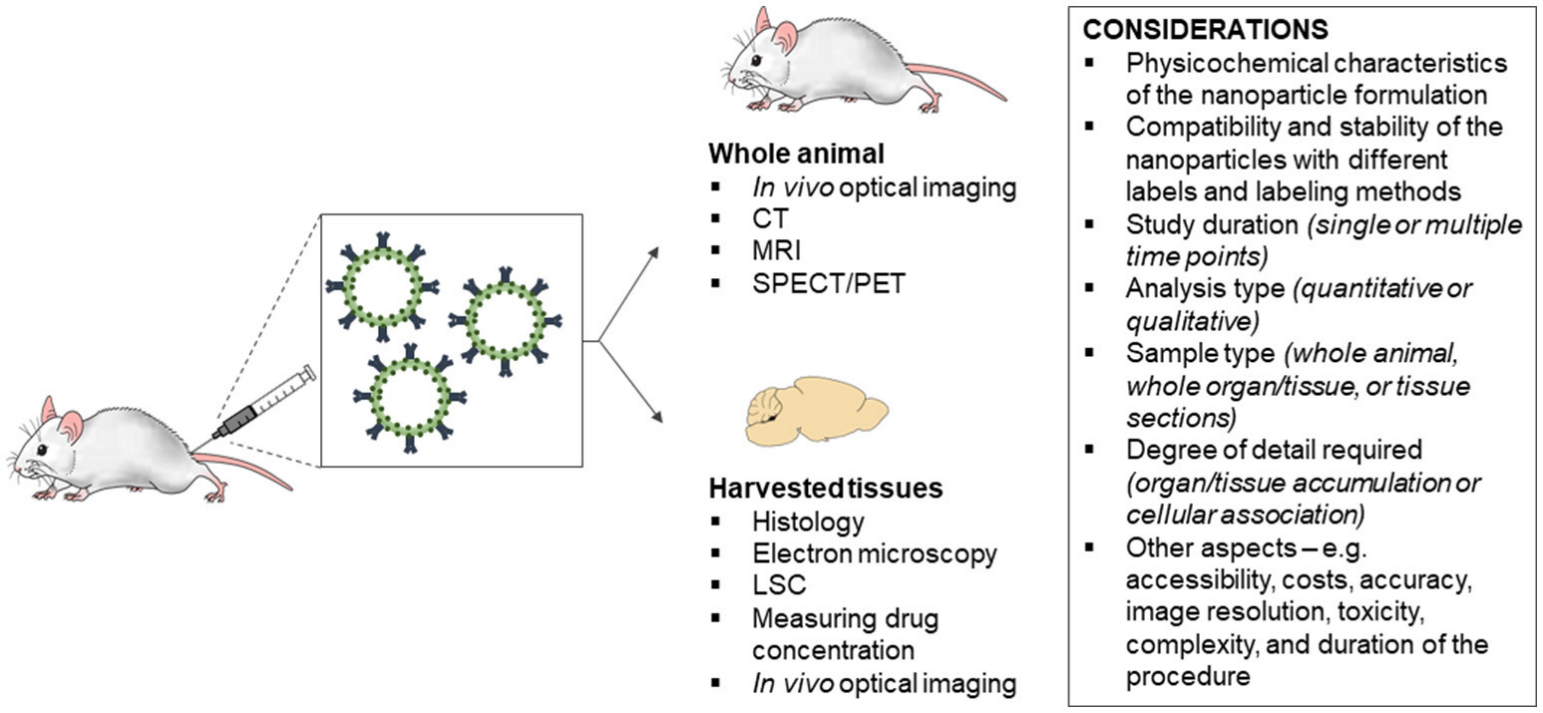
Considerations for the choice of technique for evaluating the *in vivo* biodistribution of nanoparticles. CT, computed tomography; MRI, magnetic resonance imaging; SPECT, single photon emission computed tomography; PET, positron emission tomography; LSC, liquid scintillation counting.

A key component in the translational assessment of nanomedicines is determining the biodistribution of the nanoparticles following *in vivo* administration in animals and humans (Kunjachan et al., 2015). There is a range of techniques available for evaluating nanoparticle biodistribution, including histology, electron microscopy, liquid scintillation counting (LSC), indirectly measuring drug concentrations, *in vivo* optical imaging, computed tomography (CT), magnetic resonance imaging (MRI), and nuclear medicine imaging. Each technique has its own advantages and limitations, as well as capabilities for assessing real-time, whole-organ and cellular accumulation (Figure 1). This review will address the principles and methodology of each technique and their advantages and limitations for evaluating *in vivo* biodistribution of nanoparticles.

## Histology

Microscopic visualization of nanoparticles in tissue sections is one of the common techniques used to assess biodistribution following *in vivo* administration in animals. This technique relies on histological processing of tissues to examine the association of nanoparticles with the cellular microenvironment under a microscope—typically light and fluorescence microscopy. In order to assess nanoparticle biodistribution, the organs or tissues of interest are harvested at set time points following *in vivo* administration in animals and undergo either conventional paraffin processing or cryostat processing of frozen or fixed tissues. The choice between the methods depends on the composition of the nanomedicine, as paraffin processing involves the samples being dehydrated, cleared (also called delipidation) and infiltrated. More specifically, water is removed from the specimen in successive stages using increasing concentrations of alcohol. A clearing agent, such as xylene, is used in the last phase to remove the alcohol and tissue lipids in the specimen, thereby allowing infiltration of an embedding agent (e.g., paraffin wax or cryogenic media) (Alturkistani et al., 2016). As this process involves the use of lipid soluble solvents, nanoparticles composed of materials that are easily degraded by these solvents (e.g., liposomes, solid lipid nanoparticles, and micelles) should only undergo cryostat processing of frozen sections.

For paraffin processing, tissues of interest are immediately fixed with chemicals (commonly formalin) to preserve structural integrity and prevent cell degradation (autolysis and putrefaction), prior to embedding and sectioning using a microtome. This process, however, can damage proteins in the tissue and can also denature them to a certain extent, which may include protein or peptide-based ligands conjugated to the surface of nanoparticles (Titford, 2009). On the other hand, frozen tissues may be fixed or unfixed and are sectioned using a microtome mounted in a refrigeration device known as a cryostat. Sections are then mounted on a glass slide and can be stained to enhance visualization of the nanoparticles and/or microscopic anatomy of cells and tissues. A variety of histological stains can be used to differentiate between biological structures, with hematoxylin and eosin (H&E) being the most common (Titford, 2009; Alturkistani et al., 2016). H&E staining provides excellent visualization of nuclei (stained purple) and cytoplasmic details (stained pink) within cells (Titford, 2009; Alturkistani et al., 2016). It is important to choose a stain to label biological structures that will not affect the nanoparticles themselves. Immunohistochemistry or co-labeling can also be used to visualize other aspects of the tissues, such as blood vessels, to allow appropriate orientation and evaluation of the cellular biodistribution of nanoparticles in tissue sections.

Conventional histopathology staining methods can be used to detect the biodistribution of certain types of nanoparticles. In particular, clusters of nanoparticles >200 nm in size can be visualized by light microscopy in tissue sections based on resolution limitations (Ostrowski et al., 2015; Robson et al., 2018). For example, the biodistribution of ultrasmall and small superparamagnetic iron oxide (USPIO and SPIO, respectively) nanoparticles that were injected intraperitoneally into C57BL/6 mice were studied histologically by measuring iron-positive areas (μm^2^) in representative paraffin-embedded tissue sections of organs stained with Prussian blue (Tsuchiya et al., 2011; Pham et al., 2018). Similarly, cationic stains such as Alcian blue have been used to stain the negatively charged sulfate groups embedded within organic dendritic polyglycerol sulfate (dPGS) nanoparticles. Holzhausen et al were able to demonstrate specific localization of dPGS nanoparticles in hepatic Kupffer cells following intravenous injection in mice using Alcian blue in standard histopathological tissue sections (Holzhausen et al., 2013). Single-walled carbon nanotubes (SWCNT) labeled with colloidal gold have also been visualized as dark deposits on cryostat tissue sections using silver enhancement (Mercer et al., 2008). In addition, the biodistribution of a range of nanoparticles labeled with fluorescent dyes have been visualized in tissues sections using fluorescence microscopy, including silica nanoparticles (Cho et al., 2009) and polymeric micelles (Asem et al., 2016).

In terms of advantages, histology is a relatively cost-effective technique for assessing nanoparticle biodistribution and allows for the study of large tissue sections (Table 1). In comparison to other available techniques, histology can be used to study the specific accumulation and association of nanoparticles within a cellular context. This technique also does not require exposure to ionizing radiation or contrast agents. However, histology is generally considered a qualitative method when assessing nanoparticle biodistribution and a number of limitations should be considered when approaching this technique. Light and fluorescence microscopy have generally low resolution compared to other microscopy techniques and are unable to image individual nanoparticles in the lower nanometer range, especially in tissues (Robson et al., 2018). In addition, a limited number of tissue sections (5–50 μm thickness) are typically chosen to evaluate and approximate biodistribution in each organ, simply due to the sheer number of tissue sections that can be attained from each organ. This may affect the results as not all sections are examined; therefore, appropriate sampling methods should be utilized to provide a more reliable representation of nanoparticle biodistribution in the whole organ. Histology is also a time-consuming and laborious technique. Although cryostat sectioning may be faster to prepare than paraffin-embedded tissue sections, the freezing process may negatively affect tissue structures and resolution, especially when using light microscopy. Furthermore, histology is susceptible to human error during slide preparation and analysis, and the identification of specific cell types can be difficult. In particular, the detection of organic nanoparticles in tissue sections often poses a particular challenge due to their closer similarities with biomolecules (Holzhausen et al., 2013). With regards to fluorescence imaging, the labeling of nanoparticles with fluorescent dyes may affect their physicochemical properties and subsequent *in vivo* behavior (Robson et al., 2018). Photobleaching of fluorescent dyes is another concern (Robson et al., 2018), especially when the fluorescent-labeled nanoparticles are likely to have some exposure to light during the study from *in vivo* administration to tissue harvesting and processing. This can result in a diminished fluorescent signal. Tissue autofluorescence is also a significant issue that needs to be addressed with appropriate control groups if using this technique. Autofluorescence occurs in most tissues and leads to a reduction in the signal detection sensitivity, which interferes with the accuracy of the results (Koo et al., 2006).

**Table 1:** Summary of current techniques for analyzing the biodistribution of nanoparticles.

**Technique**	**Advantages and limitations for the evaluation of nanoparticle biodistribution**
Histology	**ADVANTAGES** Relatively cost-effective technique Generally considered a qualitative method of biodistribution Allows for the study of large tissue sections Can be used to study the specific cellular association of nanoparticles within tissues Does not require exposure to ionizing radiation or contrast agents
	**DISADVANTAGES** Light and fluorescence microscopy provide low resolution imaging of nanoparticles in tissue sections Nanoparticle biodistribution in a whole organ is typically approximated by evaluating a limited number of tissue sections Time-consuming and laborious technique Freezing process for cryostat sectioning may affect tissue structure and resolution, especially when using light microscopy Susceptible to human error during slide preparation and analysis Identification and differentiation between certain cell types and nanoparticles in tissue sections can be difficult Labeling of nanoparticles with fluorescent dyes for fluorescence imaging of histology sections may affect their physicochemical properties and subsequent *in vivo* behavior Photobleaching of fluorescent-labeled nanoparticles can occur following exposure to light during *in vivo* administration to tissue harvesting and processing Tissue autofluorescence is a significant issue that needs to be addressed with appropriate control groups if using fluorescence imaging
Electron microscopy	**ADVANTAGES** Can provide detailed information of the biodistribution of nanoparticles under very high magnification Allows visualization of the accumulation of nanoparticles in cells and the localization of nanoparticles in cellular organelle Generally considered a semi-quantitative method Predominantly been used to determine the cellular association of nanoparticles *in vitro*, with only limited studies using this technique to evaluate nanoparticle biodistribution following *in vivo* administration
	**DISADVANTAGES** More expensive technique than standard histology Not capable of evaluating large tissue sections Time-consuming technique Nanoparticle biodistribution in a whole organ is typically approximated by evaluating a limited number of ultra-thin tissue sections Relatively high numbers of nanoparticles need to be administered An additional identification technique may also be necessary for a positive identification of the nanomaterial in tissues and cells Characterization of soft materials can be affected by the high-voltage electron beams Burn-in spots can form on the image to create artifacts Sample preparation method will not be suitable for all nanoparticles
Liquid scintillation counting (LSC)	**ADVANTAGES** Sensitive, specific and quantitative technique LSC can determine nanoparticle biodistribution at the tissue or organ level
	**DISADVANTAGES** This technique can be laborious, especially with the need to treat and solubilize the harvested tissues prior to LSC analysis May not be an accurate reflection of whole organ biodistribution if a small portion of an organ is collected for LSC LSC does not provide any information regarding specific cellular association or accumulation of nanoparticles in tissues Quality and reproducibility of the data will depend on the choice of the cocktail as well as on the sample composition, volume, temperature, and counting device
Measurement of drug concentration in tissues	**ADVANTAGES** Quantitative measure of biodistribution that can be used to analyze whole or partial tissue samples. Can be useful as a secondary quantitative measure to support the biodistribution results attained from qualitative techniques Does not involve exposure to ionizing radiation, incorporation of imaging molecules to nanoparticles, or the administration of contrast agents to enhance imaging outcomes
	**DISADVANTAGES** This is an indirect technique that more specifically measures payload biodistribution and may provide unreliable results if the compound prematurely dissociates from the nanoparticles following *in vivo* administration Accurate measurement of drug concentration is highly dependent upon the quality of the tissue preparation and extraction procedure, which can be time-consuming and laborious Unable to provide information on real-time biodistribution across time points in animals
*In vivo* optical imaging	**ADVANTAGES** Direct and non-invasive technique that is relatively simple to conduct Fast image acquisition times Does not require exposure to ionizing radiation Imaging can be performed in real-time and over multiple time points Can determine nanoparticle biodistribution at the tissue or organ level Images produced tend to have high sensitivity and enhanced spatial and temporal resolution Generally considered a qualitative measure of biodistribution
	**DISADVANTAGES** Can have limited tissue penetration (<1 cm) and is prone to attenuation with increased tissue depth Relatively low spatial resolution compared to CT and MRI Labeling of nanoparticles with fluorophores may alter their physicochemical properties and *in vivo* behavior Many fluorophores can undergo photobleaching during the procedure, which affects their sensitivity to imaging Tissue autofluorescence is a significant issue that can affect the interpretation of results, therefore fluorophores should have higher signal-to-background ratios Does not provide any information regarding specific cellular association or accumulation of nanoparticles in tissues It cannot visualize individual nanoparticles, but instead measures broader fluorescence intensity
Computed tomography (CT)	**ADVANTAGES** Produces reliable and high-resolution images for assessing the biodistribution of nanoparticles It has no tissue penetration limits and relatively quick image acquisition times Generally considered a qualitative measure of biodistribution Can determine nanoparticle biodistribution at the tissue or organ level Biodistribution of nanoparticles can be assessed in real-time and over multiple time points
	**DISADVANTAGES** Requires exposure to ionizing radiation Does not provide any information regarding specific cellular association of nanoparticles Often requires the administration of contrast imaging agents to enhance visualization and differentiation among different types of tissues Potential interference when nanoparticles labeled with contrast agents are used in conjunction with other contrast imaging agents to improve anatomical and tissue imaging The detection limit of nanoparticle contrast agents is less sensitive compared to other modalities, such as nuclear imaging Incorporation of contrast agents in nanoparticles may alter their physicochemical properties and *in vivo* behavior
Magnetic resonance imaging (MRI)	**ADVANTAGES** Direct and non-invasive technique Does not involve exposure to ionizing radiation Produces high spatial resolution images compared to other techniques such as optical or radionuclide imaging Provides better soft tissue contrast than CT and can differentiate better between fat, water, muscle, and soft tissue Not limited by tissue depth (unlimited penetration) Can determine nanoparticle biodistribution at the tissue or organ level Biodistribution of nanoparticles can be assessed in real-time and over multiple time points
	**DISADVANTAGES** Relatively more costly technique Has slow image acquisition and long post-processing times Much higher amount of contrast agents are generally required, as this technique can suffer from poor sensitivity Cannot be used in subjects with metallic implants/devices Incorporation of contrast agents in nanoparticles may alter their physicochemical properties and *in vivo* behavior
Nuclear medicine imaging (PET and SPECT)	**ADVANTAGES** Quantitative measure of biodistribution Biodistribution of nanoparticles can be assessed in real-time Able to image biochemical processes Not restricted by tissue penetration limits Highly sensitive technique requiring very small amounts of radiolabels, which minimizes the disruption of cell function and surrounding tissue PET is much more sensitive than SPECT and provides more radiation event localization data PET is able to substitute positron-emitters for naturally occurring atoms, thereby enhancing its ability to image molecular events SPECT can image multiple radionuclide probes simultaneously and is more widely available SPECT scans are significantly less expensive than PET scans, partly because their radionuclides are simple to prepare, more easily obtained, and typically possess longer half-lives than PET radionuclides
	**DISADVANTAGES** Relatively more costly technique Requires exposure to ionizing radiation Has slow image acquisition times Unable to be used for longitudinal studies due to radiolabel decay Has low spatial resolution and provides a lack of anatomic information, therefore it is often combined with other imaging modalities such as MRI or CT Type of radionuclide and radiolabeling strategy requires careful consideration, as some nanoparticles may exhibit differing compatibility and imaging effectiveness across the various methods SPECT has low photon detection efficiency and relatively low resolution compared to PET PET typically requires a cyclotron or generator

## Electron microscopy

Electron microscopy analysis of tissue samples can provide detailed information of the biodistribution of nanoparticles under very high magnification (Mayhew et al., 2009). This technique uses a beam of electrons focused onto the surface of the sample by various electromagnetic lenses. The electrons are scattered by the sample and are then refocused and magnified by a further series of electromagnetic lenses in the imaging column to produce a projected image (Mayhew et al., 2009; Robson et al., 2018). There are a number of different types of electron microscopes, with transmission electron microscopy (TEM), scanning electron microscopy (SEM) and variations of the two techniques having been utilized for this application. In comparison to TEM, in which the electron beam crosses the sample where it is then focused by the objective lens to form an image, SEM utilizes an electron beam that is scanned across or over a sample (rather than through a sample) and imaging is performed by mapping signal intensity synchronously with the scan to produce a magnified image of an object (Garcia-Negrete et al., 2015; Robson et al., 2018). Typically, tissue samples are fixed with chemicals (commonly formalin) and then undergo dehydration with serial alcohol and propylene oxide, prior to embedment in embedding resin (e.g., glycidether 100, EPON 812, Embed 812).

Electron microscopy has predominantly been used to determine the cellular association of nanoparticles *in vitro* (Schrand et al., 2010; Plascencia-Villa et al., 2012; Brown and Hondow, 2013; Goldstein et al., 2014), with only limited studies using this technique to evaluate nanoparticle biodistribution following *in vivo* administration (Muhlfeld et al., 2007; Mayhew et al., 2009; Jong et al., 2010; Kempen et al., 2013; Garcia-Negrete et al., 2015). For example, Jong et al. (2010) evaluated the biodistribution of gold nanoparticles (10 and 250 nm) 24 h post-intravenous injection in rats using TEM. Ultra-thin sections of 50–70 nm were prepared and stained by uranyl acetate and lead citrate. Results showed that 10 nm gold nanoparticles were present in the phagocytic cells of the reticuloendothelial system (RES), whereas 250 nm gold nanoparticles were unable to be detected in any of the organs investigated. This was likely due to the very low number of 250 nm particles that would be theoretically present in one TEM tissue section. In addition, several globular structures of approximately the expected size were found in liver cells and the endothelium of blood vessels in the brain; however, elemental analysis with energy dispersive X-ray (EDX) showed that these structures did not contain gold. This indicates that *in vivo* identification of nanoparticles cannot only depend on the detection of nanosized structures in cells.

More recent studies have used scanning transmission electron microscopy (STEM), which combines the principles of TEM and SEM (Kempen et al., 2013; Garcia-Negrete et al., 2015). STEM requires very thin samples (similar to TEM) and involves scanning a very finely focused beam of electrons across the sample in a raster pattern. This technique allows the use of other signals that cannot be spatially correlated in TEM (e.g., secondary electrons, scattered beam electrons, characteristic X-rays, and electron energy loss) and has improved spatial resolution compared to SEM (Kempen et al., 2013; Garcia-Negrete et al., 2015). For example, Kempen et al. (2013) used STEM to analyze the accumulation and distribution of polyethylene glycol coated Raman-active-silica-gold-nanoparticles (PEG-R-Si-Au-NPs) in the liver of intrarectally administered or tail-vein injected mice. Tissue samples were trimmed to <1 mm^3^ and prepared and stained by osmium tetroxide and uranyl acetate. Sections (150 nm thick) were cut from the block face using an ultramicrotome and then placed on a copper grid. This approach utilizes the simultaneous bright and dark field imaging capabilities of STEM to readily identify PEG-R-Si-Au-NPs in mouse liver tissue. Results showed that nanoparticles injected intravenously accumulated in the liver while those administered intrarectally did not, indicating that they remain in the colon and do not pass through the colon wall into the systemic circulation.

Overall, the main advantage with electron microscopy is the high resolution, which allows visualization of the accumulation of nanoparticles in cells and the localization of nanoparticles in cellular organelles (Jong et al., 2010). Although this technique is generally considered a semi-quantitative method, a number of limitations should be considered when approaching this technique for evaluating nanoparticle biodistribution. Electron microscopy is a more expensive technique and is not capable of evaluating large tissue sections compared to standard histology (Table 1). For example, the analysis volume for TEM is generally low at 1–10 μm^3^ for a single TEM session (Kempen et al., 2013). In addition, a limited number of ultra-thin tissue sections (50–150 nm thickness) are typically chosen to evaluate and approximate biodistribution in each organ, which may affect the results as not all sections are examined. Therefore, appropriate sampling methods and additional analytical methods should be utilized to provide a more reliable representation of nanoparticle biodistribution in the whole organ. Electron microscopy is also a time-consuming technique, with individual samples usually taking >3–4 h to analyze (Kempen et al., 2013). Relatively high numbers of nanoparticles need to be administered to enable the detection of nanoparticles in organs by electron microscopy, especially for larger nanoparticles (Jong et al., 2010; Kempen et al., 2013). An additional identification technique (e.g., EDX detection of the composing elements or a specific marker for the administered nanoparticles) may also be necessary for a positive identification of the nanomaterial in tissues and cells (Jong et al., 2010). Although electron microscopy can readily image soft matter samples, characterization of soft materials can be affected by the high-voltage electron beams (Garcia-Negrete et al., 2015). Therefore, artifacts need to be carefully protected against when an image is acquired, as burn-in spots can form on the image (Kempen et al., 2013). With regards to radiation exposure, low levels of X-rays can be produced from the backscattered electrons impinging on samples in electron microscopes. However, these units are well-shielded and any X-rays generated internally should not penetrate outside the unit. Finally, the sample preparation method will not be suitable for all nanoparticles. As this process involves the use of lipid soluble solvents, nanoparticles composed of materials that are easily degraded by these solvents (e.g., liposomes, solid lipid nanoparticles and micelles) should use other biodistribution techniques.

## Liquid scintillation counting (LSC)

Liquid scintillation counting (LSC) is a standard laboratory method to quantify the radioactivity of low energy radioisotopes—most commonly beta-emitting (β-emitting) and alpha-emitting (α-emitting) isotopes (Shigematsu et al., 1995; PerkinElmer, 2008). LSC analysis of samples requires a specific cocktail containing the aromatic organic solvent and scintillators (also referred to as “fluors”) to absorb the radioisotopic energy and produce detectable light pulses, respectively. The basic principles of LSC rely on the energy released from a radioactive decay (emitting beta or alpha particles) to excite the aromatic solvent molecules. The energy of the solvent molecules is then transferred to the scintillator molecules to produce excited states of the electrons, which decay to the ground state and produce a light pulse that is characteristic for the scintillator. The emitted light is detected by the photomultiplier tube (PMT) of the liquid scintillation counter.

*In vivo* biodistribution of nanomedicines can be assessed by labeling nanoparticles with isotopic markers prior to administration in animals. For example, anti-ICAM-1 immunoliposomes and control liposomes were radiolabeled with [^3^H]-CHE and administered intravenously in rats with Complete Freund's Adjuvant-induced inflammation of the paw (Hua and Cabot, 2013). The use of [^3^H]-CHE is convenient for these studies because it is a stable, non-exchangeable, and non-degradable marker of liposomes, thus providing an estimate of the cumulative liposome dose in tissues (Hua and Cabot, 2013). Organs are then harvested and prepared for LSC. Depending on the sample type, the biological material can either be directly mixed to the cocktail with no or little pre-treatment, or a treatment/solubilization may be needed prior to scintillation cocktail addition (PerkinElmer, 2008). The latter is generally required when analyzing biological tissues and usually a portion of an organ is weighed and processed, due to the time taken for effective solubilization of larger tissues. Radioactivity is measured in terms of number of disintegrations per minute (DPM) of the isotope in each tissue sample. The amount of radioactivity can then be expressed as the number of becquerel (Bq) per gram of tissue using the following conversion: 1 Bq = 60 DPM. The becquerel is the SI derived unit of radioactivity.

LSC has the advantages of being a sensitive, specific, and quantitative technique for measuring nanomedicine biodistribution (Table 1). Removal of excess free isotopic markers that have not been incorporated into the nanoparticles is important prior to *in vivo* assessment. It should be noted that the quality and reproducibility of the data will depend on the choice of the cocktail as well as on the sample composition, volume, temperature, and counting device (PerkinElmer, 2008). This technique can be laborious, especially with the need to treat and solubilize the harvested tissues prior to LSC analysis. If a small portion of an organ is collected for LSC, this may not be an accurate reflection of whole organ biodistribution. Furthermore, LSC can only determine nanoparticle biodistribution at the tissue or organ level and does not provide any information regarding specific cellular association or accumulation of nanoparticles in tissues.

## Measurement of drug concentration in tissues

Nanoparticles loaded with therapeutic compounds can have their biodistribution evaluated by measuring drug concentration in tissues. This is an indirect approach and more specifically determines payload biodistribution. The assumption is that nanoparticles accumulate in specific tissues following *in vivo* administration, where they then release their cargo. It does not take into account possible premature drug release from the nanoparticles into the circulation and subsequent biodistribution of the free drugs themselves. This technique involves tissue samples being prepared for solubilization and extraction of the specific compound for further analysis. In order to achieve effective drug extraction from tissues, it is important to first determine the physicochemical properties of the compound and tissue matrix in the sample (Pavlović et al., 2007).

In brief, biological tissues are broken down by methods such as grinding, blending, homogenization, sonication or sieving, as finer samples are more homogenous and easier to extract. Particulates are removed from the coarse biological material through methods such as centrifugation, filtration or solid-phase extraction. The supernatant is then collected and subjected to further extraction and purification. The extraction of drugs from biological tissues depends on its physicochemical properties, such as solubility, hydrophobicity/hydrophilicity, ionization, partition coefficient, and molecular weight. For example, solid-liquid extraction may be used, where a solvent is added to dissolve the analyte in the sample. The mixture is then filtered, decanted, or centrifuged to separate the solvent from the remaining sample. Following extraction, evaporation and reconstitution may be required before final analysis with high-performance liquid chromatography (HPLC) and/or mass spectrometry (MS) (Majors, 2013).

Measurement of drug concentration in tissues has been widely used for determining the biodistribution of nanomedicines. For example, Milane et al. (2011) assessed the biodistribution of epidermal growth factor receptor (EGFR)-targeted polymer-blend nanoparticles loaded with the anti-cancer drugs, lonidamine and paclitaxel, in an orthotopic animal model of multi-drug resistant breast cancer. After euthanasia, the tumor mass, liver, lungs, kidneys, spleen, and heart were harvested and weighed. Tissue and plasma samples were then prepared using established methods for the extraction of lonidamine and paclitaxel in preparation for HPLC analysis. The data showed that both the non-targeted and the targeted nanoparticles were effective at increasing the tumor concentration of paclitaxel and lonidamine relative to free drug solution.

The main advantage of this technique is that it provides a quantitative measure of biodistribution that can be used to analyze whole or partial tissue samples (Table 1). This method does not involve exposure to ionizing radiation, incorporation of imaging molecules to nanoparticles, or the administration of contrast agents to enhance imaging outcomes. However, as mentioned earlier this indirect technique more specifically measures payload biodistribution. It is the compound encapsulated into or incorporated on the surface of the nanoparticles that is measured, which may provide unreliable results if the compound prematurely dissociates from the nanoparticles following *in vivo* administration. Furthermore, accurate measurement of drug concentration is highly dependent upon the quality of the tissue preparation and extraction procedure, which can be time-consuming and laborious. This technique is also unable to provide information on real-time biodistribution across time points in animals, but can be used as a secondary quantitative measure to support the biodistribution results attained from qualitative techniques.

## *In vivo* optical imaging

This technique refers to the use of equipment such as the *In Vivo* Imaging System (IVIS®) and Kodak *In-Vivo* FX Imaging Station to visualize the biodistribution of nanoparticles in real-time in live animals or in harvested tissues and organs. These *in vivo* imaging systems are non-invasive and involve optical imaging technology to evaluate fluorescence or bioluminescence within the sample. Even though *in vivo* imaging systems typically possess these dual imaging capabilities, fluorescence imaging is used the most to evaluate the biodistribution of nanoparticles. Fluorescent imaging employs the ability of fluorophores, such as fluorescent proteins, dyes and conjugated polymers, to fluoresce after being excited with light of a particular wavelength (Janib et al., 2010; Coll, 2011; Priem et al., 2015). Fluorophores can be encapsulated within the nanoparticles (core or membrane) or conjugated to the nanoparticle surface. To optimize *in vivo* imaging sensitivity, fluorescent contrast agents should emit light in the red or near infrared (near-IR) wavelengths (~600–1,000 nm) (Coll, 2011; Liu Y. et al., 2012). This is particularly important for deep tissue samples to avoid coinciding with low photon absorption and autofluorescence in tissues, thereby enabling higher signal-to-background ratios (Vats et al., 2017). Once the sample is excited by a light source within the imaging chamber, fluorescence is emitted and captured on a charge-coupled device (CCD) camera that then converts this into electrical signals (Coll, 2011). A three-dimensional, tomographic image depicting the biodistribution of the fluorescent probe is then reconstructured.

*In vivo* imaging systems are commonly used to evaluate the biodistribution of nanoparticles, particularly in live animals across various time points to assess accumulation relative to disease progression. A variety of fluorescent-labeled nanoparticles have been imaged using this technique, including nanoporous silicon nanoparticles, carbon nanotubes, metal-based nanoparticles, polymer-based nanoparticles, and lipid-based nanoparticles (Connell et al., 2002; Zheng et al., 2003; Gao et al., 2010b; Goldberg et al., 2011; Milane et al., 2011; Tasciotti et al., 2011; Liu Y. et al., 2012; Zhang et al., 2012). For example, Milane et al. (2011) used this technique as a qualitative assessment of the biodistribution of EGFR-targeted polymer-blend nanoparticles in an orthotopic animal model of multi-drug resistant breast cancer. In this study, non-targeted and targeted nanoparticles loaded with DiR (near-IR) dye were administered via tail vein injection, and the biodistribution was visualized using a Kodak *In-Vivo* FX Imaging Station over 6 h. The results attained from *in vivo* optical imaging were found to be comparable with the quantitative data attained from HPLC analysis of drug distribution. Interestingly, some nanoparticles possess contrast that is inherently fluorescent such as quantum dot nanocrystals (Gao et al., 2010a,b; Liu Y. et al., 2012; Zhang et al., 2012; Zhao and Zeng, 2015). Quantum dots are semiconductor nanocrystals synthesized with a core-shell structure that enables imaging in the near infrared spectrum, thereby enhancing image sensitivity. They possess attractive optical qualities such as size-tunable fluorescence, photostability, high fluorescence quantum yields, and high resistance to photobleaching (Gao et al., 2010a). However, quantum dot preparations contain heavy metals such as cadmium, tellurium and selenium, which are potentially toxic to the body (Hardman, 2006; Kim et al., 2017).

Overall, *in vivo* optical imaging has the advantages of being direct, non-invasive and relatively simple to conduct (Table 1). It has fast image acquisition times and the procedure does not require exposure to ionizing radiation (Koo et al., 2006; Liu Y. et al., 2012). As imaging can be performed in real-time, biodistribution of nanoparticles can be assessed over many time points in the same group of animals—thus allowing a reduction in animal numbers. The images produced tend to have high sensitivity and enhanced temporal resolution (Liu Y. et al., 2012; Kim et al., 2017). This technique is generally considered a qualitative measure of biodistribution, as the intensity measured is not necessarily relative to the number of nanoparticles present in the tissues (Liu Y. et al., 2012). There are also a few limitations to this technique that should be considered. *In vivo* imaging systems can have limited tissue penetration (<1 cm) and is prone to attenuation with increased tissue depth (Koo et al., 2006; Kim et al., 2017). This is due to interference from light absorption and light scattering by tissue biomatter. This technique also has relatively low spatial resolution compared to CT and MRI (Massoud and Gambhir, 2003). In addition, labeling of nanoparticles with fluorophores may alter their physicochemical properties (e.g., surface charge, size, and surface functionalization) and *in vivo* behavior (Ann et al., 2013; Robson et al., 2018). Therefore, the choice of fluorophore and the method for labeling nanoparticles should be carefully considered. Another concern is that many fluorophores can undergo photobleaching (Robson et al., 2018), which affects their sensitivity to imaging. Tissue autofluorescence is a significant issue that can affect the interpretation of results, therefore fluorophores should have high signal-to-background ratios (Koo et al., 2006). Furthermore, *in vivo* imaging systems can only determine nanoparticle biodistribution at the tissue or organ level and do not provide any information regarding specific cellular association or accumulation of nanoparticles in tissues. It cannot visualize individual nanoparticles, but instead measures broader fluorescence intensity.

## Computed tomography (CT)

Computed tomography (CT) is a non-invasive, radiological imaging technique that uses X-rays to produce three-dimensional, tomographic (cross-sectional) images of tissues. This technique is based on the variable absorption of X-rays by different tissues, which is a form of ionizing radiation with wavelengths of ~0.01–10 nm (Kim et al., 2017). CT scanners typically consist of an X-ray tube, a detector unit, an image reconstruction system, collimators and filters. The X-ray tube is composed of a cathode and a tungsten-alloy anode housed within a vacuum. X-rays are generated within the tube by applying high voltage, which accelerates electrons from the heated cathode filament toward the anode. The accelerated electrons interact with electrons of the anode's tungsten nuclei and subsequently cause emission of X-rays. X-rays are then passed through the subject and are attenuated (absorbed or scattered), resulting in a loss of X-ray intensity (Lusic and Grinstaff, 2013; Liguori et al., 2015; Kim et al., 2017). Differential attenuation of X-rays across tissues according to their attenuation coefficient causes variation in radiation intensities and depicts information about tissue density and structure (Chatterjee et al., 2014; Liguori et al., 2015). This information is captured by detectors as a series of projections. Usually, the X-ray tube and detectors rotate synchronously on a circular axis around the subject with detectors positioned directly opposite, which enables a complete dataset of projections to be obtained over 360°. Computer algorithms are then applied to produce a three-dimensional reconstruction of the scanned object. Collimators and filters are used to limit unwarranted radiation and enhance the quality of the image (Liguori et al., 2015).

Contrast within the final image depends on the different densities and thickness of body structures. While different types of tissues can exhibit contrast, it can be particularly challenging to achieve high quality images and identify the interface between two different adjacent tissues (e.g., tumor in an organ) or to image soft tissues in contact with bodily fluids (Lusic and Grinstaff, 2013; Chatterjee et al., 2014). Therefore, contrast imaging agents are often used to increase CT sensitivity to enhance visualization and differentiation among different tissues. Contrast agents are usually elements having high atomic numbers and, therefore, higher number of electrons, which attenuate X-rays more efficiently by absorbing external X-rays. This results in decreased exposure on the X-ray detector (Lusic and Grinstaff, 2013). Contrast agents used clinically in patients undergoing CT are typically iodine- or barium-based compounds. Iodinated contrast agents are the main type of radiocontrast used for vascular imaging (e.g., vascular calcifications and hemorrhage), whereas barium sulfate is mainly used for imaging the gastrointestinal tract (Lusic and Grinstaff, 2013; Chatterjee et al., 2014; Kim et al., 2017).

CT has been utilized as a technique to allow *in vivo* imaging of the biodistribution of nanoparticles in real-time. Electron-dense elements are typically incorporated into the nanoparticles to enable visualization and differentiation of the nanoparticles in the tissues. Contrast agents that are more commonly incorporated into nanoparticles for CT analysis include iodine (Torchilin et al., 1999; Yordanov et al., 2002; Fu et al., 2006; Ho Kong et al., 2007; Elrod et al., 2009; de Vries et al., 2010; Hill et al., 2010; Hallouard et al., 2011), gold (Chie et al., 2010; Guo et al., 2010; Wang et al., 2011; Xiao et al., 2013), and bismuth (Rabin et al., 2006; Naha et al., 2014). However, various other elements such as gadolinium (Zhou et al., 2014), platinum (Chou et al., 2010), tantalum (Bonitatibus et al., 2010; Oh et al., 2011), tungsten (Jakhmola et al., 2014; Firouzi et al., 2017), and ytterbium (Pan et al., 2012; Jianhua et al., 2013) have also been used. Contrast agents for CT imaging can be loaded into the core of the nanoparticles, chemically grafted to the surface of nanoparticles, or inserted into the carrier membrane (e.g., lipid bilayer) (Cormode et al., 2014; Li et al., 2014). The *in vivo* biodistribution of numerous types of nanoparticles have been studied with CT, including nano-emulsions (de Vries et al., 2010; Hallouard et al., 2011), liposomes (Sachse et al., 1997; Leander et al., 2001; Elrod et al., 2009), micelles (Torchilin et al., 1999; Torchilin, 2002), lipoproteins (Cormode et al., 2008; Hill et al., 2010), polymer-coated nanoparticles (Rabin et al., 2006; Muddineti et al., 2015; Firouzi et al., 2017), nanocapsules/nanospheres (Ashcroft et al., 2007; Ho Kong et al., 2007), nanotubes/nanorods (Ashcroft et al., 2007; Zhou et al., 2014), metal-based nanoparticles (Bonitatibus et al., 2010; Chou et al., 2010; Oh et al., 2011; Pan et al., 2012; Jianhua et al., 2013; Mieszawska et al., 2013; Cormode et al., 2014; Jakhmola et al., 2014; Naha et al., 2014; Kim et al., 2017), and dendrimers (Yordanov et al., 2002; Fu et al., 2006; Chie et al., 2010; Guo et al., 2010; Wang et al., 2011; Xiao et al., 2013).

CT has demonstrated to be an effective technique for producing reliable and high-resolution images for assessing the biodistribution of nanoparticles (Table 1). It has no tissue penetration limits and relatively quick image acquisition times (Massoud and Gambhir, 2003). This technique is generally considered a qualitative measure of biodistribution and can only determine nanoparticle biodistribution at the tissue or organ level. Furthermore, CT requires exposure to ionizing radiation and does not provide any information regarding specific cellular association of nanoparticles (Kim et al., 2017). Biodistribution of nanoparticles can be assessed in real-time and over many time points in the same group of animals, which reduces the number of animals required for longitudinal studies. However, CT alone can suffer from relatively poor visualization and differentiation among different types of tissues as mentioned above (Lusic and Grinstaff, 2013; Chatterjee et al., 2014). Hence, it often requires the administration of contrast imaging agents to increase CT sensitivity. This can pose a problem when nanoparticles labeled with contrast agents are used in conjunction with other contrast imaging agents to improve anatomical and tissue imaging. The detection limit of nanoparticle contrast agents is less sensitive compared to other modalities, such as nuclear imaging (Massoud and Gambhir, 2003; Kim et al., 2017). To overcome this issue, nanoparticles incorporating high concentrations of contrast agents are often required to improve imaging. Incorporation of contrast agents in nanoparticles may alter their physicochemical properties and *in vivo* behavior (Massoud and Gambhir, 2003; Kim et al., 2017).

## Magnetic resonance imaging (MRI)

Magnetic resonance imaging (MRI) is a non-invasive imaging technique that produces three dimensional detailed anatomical images, without the use of ionizing radiation. MRI uses powerful magnets that produce a strong magnetic field that forces protons in the body to align with that field (Strijkers et al., 2007; Grover et al., 2015). Protons (hydrogen nuclei) are typically used in MRI imaging as they are particularly abundant in the water and fat of the body. Protons possess a positive charge and are constantly spinning around their own axes, which generates a magnetic field. The magnetic field for each proton is known as a magnetic moment and is a measure of an object's tendency to align with a magnetic field. Radiofrequency currents are pulsed through the patient to excite the protons to a higher energy state and spin them out of equilibrium, which creates strain against the pull of the magnetic field (Grover et al., 2015). When the radiofrequency field is turned off, the protons then realign with the magnetic field and the MRI sensors can detect the energy that is released in this process. In particular, MRI is able to produce high-resolution images by measuring the spin magnetization of polarized protons and their respective longitudinal (T_1_) and transverse (T_2_) relaxation rates in the body. It utilizes magnetic fields, electric field gradients and radio waves to produce three types of images: spin density weighted, T_1_ weighted and T_2_ weighted images (Strijkers et al., 2007; Grover et al., 2015). Field gradient coils are used to localize the MRI signal to particular tissues of interest. The signals are processed to extract frequency and phase data, and a mathematical algorithm is applied to construct an image. The time it takes for the protons to realign with the magnetic field and the amount of energy released changes depending on the environment and the chemical nature of the molecules, which is used to differentiate between various types of tissues (Grover et al., 2015).

Contrast agents may be administered to a patient intravenously before or during the MRI procedure to increase the speed at which protons realign with the magnetic field, thereby shortening the T_1_ and/or T_2_ relaxation rates of protons located in their vicinity. Contrast agents that shorten T_1_ (paramagnetic contrast agents) result in T_1_ and ${\text{T}}_{1}^{*}$ hypersignal (brighter images), whereas those that shorten T_2_ (superparamagnetic contrast agents) lead to a reduction in the T_2_ and ${\text{T}}_{2}^{*}$ signal (darker images) (Strijkers et al., 2007; Kamaly and Miller, 2010). This improvement in image quality also enhances the differentiation between tissues. The effectiveness of contrast agents depends on its relaxivity, which is the proportionality constant of the measured rate of relaxation: 1/T_1_ and 1/T_2_ (Sun et al., 2008). Superparamagnetic iron oxide crystals (Fe^3+^ or Fe^2+^) and paramagnetic lanthanide metals, such as gadolinium (Gd^+3^), are the most widely used contrast agents for MRI imaging (Strijkers et al., 2007; Kamaly and Miller, 2010). Superparamagnetic iron oxide (SPIO, >50 nm in size) and ultrasmall superparamagnetic iron oxide (USPIO, < 50 nm in size) are mainly used to shorten T_2_, leading to darker images in T_2_ and ${\text{T}}_{2}^{*}$ weighted MRI (Jung and Jacobs, 1995; Strijkers et al., 2007; Kamaly and Miller, 2010). Conversely, paramagnetic gadolinium ions are used to shorten T_1_, resulting in brighter images in T_1_ weighted MRI (Strijkers et al., 2007; Kamaly and Miller, 2010). The most clinically used MRI contrast agents are those that shorten T_1_ relaxation rates, hence those that contain the element gadolinium are often preferred (Sun et al., 2008).

Gadolinium (Gd^+3^) has seven unpaired outer shell electrons and a large magnetic moment, making it extremely useful for MRI imaging (Strijkers et al., 2007; Kamaly and Miller, 2010). Free gadolinium ions are highly toxic and, therefore, they are usually chelated with other ligands (e.g., diethylenetriamine pentaacetic acid, DTPA; tetraazacyclododecane tetraacetic acid, DOTA) to form complexes that are nontoxic and highly stable in the body during the period of administration (Wiegers et al., 1992; Rosen et al., 2011). For example, Park et al conjugated the peptide RGD to Gd-DOTA to obtain an MRI contrast agent with tumor targeting capability (Park et al., 2008). One of the more common gadolinium chelate used clinically and in drug delivery is gadoteridol, which is the chelate formed between Gd^+3^ and 10-(2-hydroxy-propyl)-1,4,7,10-tetraazacyclododecane-1,4,7-triacetate (Zhou and Lu, 2013). Gadolinium-based contrast agents can be incorporated into nanoparticles to enable real-time imaging of their *in vivo* biodistribution using MRI. A number of nanoparticle types have incorporated these contrast agents, including liposomes (Unger et al., 1989; Saito et al., 2005; Hossann et al., 2013; Smith et al., 2013; Skupin-Mrugalska et al., 2018), dendrimers (Margerum et al., 1997; Lee et al., 2005; Rongzuo et al., 2007), micelles (Parac-Vogt Tatjana et al., 2004; Kumar et al., 2010), polymeric-based nanoparticles (Liu et al., 2011), carbon-based nanotubes (Hartman et al., 2008; Richard et al., 2008), and mesoporous silica nanoparticles (Kobayashi et al., 2007; Kim et al., 2008; Taylor et al., 2008). For example, Saito et al. (2005) manufactured gadoteridol-loaded liposomes for real-time MRI evaluation of convection-enhanced delivery in the primate brain. Volume of distribution was analyzed for all delivery locations by histology and MRI, following administration in the corona radiata, putamen nucleus, and brain stem. The results showed that MRI of liposomal gadolinium was highly accurate at determining tissue distribution, as confirmed by comparison with histological results from concomitant administration of fluorescent liposomes. Gadolinium-based contrast agents can be incorporated within the core of nanoparticles, attached to the particle surface, or inserted into the carrier membrane (Unger et al., 1989; Hossann et al., 2013; Smith et al., 2013; Skupin-Mrugalska et al., 2018). It should be noted that encapsulation of gadolinium within the core can lead to lowered relaxivity, whereas surface attachment may be preferable to improve gadolinium's ability to interact with water (Tilcock et al., 1989; Kamaly and Miller, 2010). Relaxivity can be further improved by reducing the size of the nanoparticles (Tilcock et al., 1989; Kamaly and Miller, 2010).

MRI can also be used to evaluate the biodistribution of nanoparticles formulated with superparamagnetic iron oxide cores. Iron oxide crystals are mainly utilized to provide negative contrast in T_2_ and ${\text{T}}_{2}^{*}$ weighted images. SPIO and USPIO nanoparticles are usually composed of a nano-sized magnetite (Fe_3_O_4_) or maghemite (γ-Fe_2_O_3_) core coated with a variety of materials to enhance stability, circulation time, biocompatibility and minimize toxicity (Peng et al., 2008). Bulk iron oxide is ferromagnetic, however when nano-sized, superparamagnetism is exhibited (Di Marco et al., 2007). The superparamagnetism of iron oxide nanoparticles is important for *in vivo* imaging. Polymers are the most widely used stabilizing materials and can be adsorbed into or anchored onto the iron oxide surface via hydrogen bonds, electrostatic forces or pseudo-covalent bonding (Estelrich et al., 2015). Examples include poly(ethylene glycol) (PEG), alginate, chitosan, dextran and its derivatives, starch, polyvinyl alcohol, albumin, poly(ethylene imine), organic siloxane, and sulphonated styrene-divinyl-benzene (Estelrich et al., 2015). In addition, SPIO can be used alone or incorporated into other nanostructures, such as magnetoliposomes (SPIOs are hybridized within a liposome carrier) (Martina et al., 2005; Plassat et al., 2007) and colloidal iron oxide nanoparticles (oleate-coated magnetite particles embedded in a hydrophobic matrix) (Senpan et al., 2009). Several formulations of iron oxide nanoparticles are already approved for clinical use (e.g., ferumoxides and ferucarbotran) for contrast-enhanced MRI of the liver (Reimer and Tombach, 1998). Their relatively large surface area also enables incorporation of biologically active substances to the surface of the nanoparticles. For example, Veiseh et al. (2009) developed a nanoprobe consisting of an iron oxide nanoparticle coated with biocompatible PEG–grafted chitosan copolymer, which allowed conjugation of a tumor-targeting agent, chlorotoxin, and a near-IR fluorophore. The results showed an ability for the nanoprobe to cross the blood-brain barrier and specifically target brain tumors in a genetically engineered mouse model, as evidenced by *in vivo* MRI evaluation, *in vivo* optimal imaging and histology. The magnetism and subsequent MRI effectiveness of iron oxide nanoparticles is dependent upon their size, shape, morphology, structure, and homogeneity (Lin et al., 2012; Estelrich et al., 2015). Thus, variations in SPIO and USPIO nanoparticles can lead to different magnetic properties and thus alter their function in various applications. The coating and surface modifications can also influence *in vivo* stability and biodistribution of the nanoparticles. It should be noted that for conventional MRI, SPIO nanoparticles give negative contrast enhancement (dark signals) that are often confounded by the presence of artifacts due to hemorrhage, air, and partial-volume effects. To address these issues, many attempts have been made to generate positive contrast visualization methods in the last decade (Lin et al., 2012; Estelrich et al., 2015).

Overall, MRI has the advantage of producing high spatial resolution images (micrometers rather than several millimeters) compared to other techniques such as optical or radionuclide imaging (Massoud and Gambhir, 2003) (Table 1). It provides better soft tissue contrast than CT and can differentiate better between fat, water, muscle, and soft tissue (Massoud and Gambhir, 2003; Janib et al., 2010). MRI is not limited by tissue depth (unlimited penetration) and does not involve exposure to ionizing radiation. Furthermore, this technique allows non-invasive, three-dimensional, real-time imaging of the biodistribution of nanoparticles *in vivo*. However, MRI is more costly and has slow image acquisition and long post-processing times (Kim et al., 2017). As this technique can suffer from poor sensitivity, much higher amounts of contrast agent generally need to be administered (Massoud and Gambhir, 2003; Kim et al., 2017). MRI cannot be used in subjects with metallic implants/devices (Janib et al., 2010). In addition, incorporation of contrast agents in nanoparticles may alter their physicochemical properties and *in vivo* behavior (Massoud and Gambhir, 2003; Kim et al., 2017).

## Nuclear medicine imaging

Single photon emission computed tomography (SPECT) and positron emission tomography (PET) scans are the two most common imaging modalities in nuclear medicine. They are both non-invasive techniques that produce three-dimensional images of the body by detecting gamma rays (γ-rays) that are emitted from radioactive substances that become localized and are taken up by specific tissues (Townsend, 2004; Ziegler, 2005; Pimlott and Sutherland, 2011; Van Audenhaege et al., 2015). Both techniques essentially involve administration of a radioactive tracer (radiotracer) into the subject that consists of a molecular probe with a radioactive isotope attached that is capable of emitting γ-rays. The choice of molecular probe is dependent on the tissue to be imaged and should ideally have high affinity and high selectivity for the target receptor or organ (Pimlott and Sutherland, 2011). As the isotope decays in the tissue, it emits gamma rays that are picked up by detectors (gamma scintillation camera system) placed around the subject. The scintillation crystals within the detectors then convert the γ-ray energy into lower-energy (near-optical) photons. This optical energy is converted into electrical signals by photomultiplier tubes and processed to obtain the location of the scintillation events in the crystal (Townsend, 2004; Ziegler, 2005; Peterson and Furenlid, 2011). The radionuclide is captured in a collection of projections, which are measured from numerous angles and linear positions in the subject. Image reconstruction techniques are applied to reconstruct these projections into a three-dimensional, tomographic image of the radiotracer's biodistribution and concentration within the tissue (Townsend, 2004; Ziegler, 2005; Peterson and Furenlid, 2011; Pimlott and Sutherland, 2011).

Although both PET and SPECT rely on the detection of gamma radiation, they differ in the type of radionuclides used. The radionuclides used in SPECT emit γ-rays by radioactive decay that is measured directly, whereas PET radionuclides emit positrons that annihilate with electrons up to a few millimeters away in the tissue to produce two gamma photons that are emitted in opposite directions (Massoud and Gambhir, 2003; Townsend, 2004; Ziegler, 2005). The γ-rays emitted in PET are captured in coincidence by opposing pairs of detectors aligned collinearly around the subject, which enable measurement of the radionuclide from multiple angles and planes (Townsend, 2004; Ziegler, 2005). Unlike PET, SPECT gamma cameras are rotated around the subject and a lead collimator is required to reconstruct the original location of the emitted γ*-*rays (Peterson and Furenlid, 2011; Van Audenhaege et al., 2015). PET positron emitters (e.g., ^15^O, ^64^Cu, ^13^N, ^11^C, and ^18^F) emit higher energy γ-rays and possess shorter radioactive half-lives than SPECT radiotracers (Massoud and Gambhir, 2003; Townsend, 2004; Ziegler, 2005). The most common radioisotopes used for SPECT imaging include ^99m^Tc, ^111^In, and radioiodine (e.g., ^131^I) (Hong et al., 2009; Pimlott and Sutherland, 2011).

Nanoparticles can be labeled with gamma-emitting radionuclides and positron emitters. These radiolabels can be attached to the nanoparticle surface, conjugated to the nanoparticle core, or encapsulated within a payload that is loaded into the nanoparticle. Radiolabeling is achieved through methods such as exogenous chelation of radiometals, direct proton/neutron bombardment, and chelator-free radiolabeling (Gibson et al., 2011; Liu T. et al., 2012 Sun et al., 2015; Lu et al., 2018; Yuan et al., 2018). Alternatively, radioactive precursors can be used to synthesize intrinsically radioactive nanoparticles (Zhao et al., 2014; Sun et al., 2015). The type of radionuclide and radiolabeling strategy requires careful consideration, as some nanoparticles may exhibit differing compatibility and imaging effectiveness across the various methods (Liu and Welch, 2012). PET can be used to image the biodistribution of a variety of nanoparticles, including quantum dots (Ducongé et al., 2008; Tu et al., 2011), iron oxide nanoparticles (Glaus et al., 2010; Yang et al., 2011), gold nanoparticles (Xie et al., 2010; Guerrero et al., 2012), liposomes (Oku et al., 2011; Petersen et al., 2011), solid lipid nanoparticles (Andreozzi et al., 2011), polymer-based nanoparticles (Fukukawa et al., 2008; Herth et al., 2009; Allmeroth et al., 2013), carbon-based nanoparticles (Liu et al., 2006; McDevitt et al., 2007), and micelles (Xiao et al., 2012). Similarly, SPECT imaging, often in combination with other imaging modalities, can also image the biodistribution of a similar range of nanoparticles, including dendrimers (Zhang et al., 2010a,b), micelles (Cheng et al., 2013; Hong et al., 2014), liposomes (Chang et al., 2010), carbon-based nanoparticles (Wu et al., 2009), iron-oxide nanoparticles (Madru et al., 2012), polymeric nanoparticles (Lu et al., 2011), gold nanoparticles (Morales-Avila et al., 2011; You et al., 2012), and silver nanoparticles (Chrastina and Schnitzer, 2010).

As PET and SPECT imaging rely purely on the detection of γ*-*rays, radiolabels must remain attached to the nanoparticles to accurately image their biodistribution. If disassociation occurs, imaging will not reflect true biodistribution, resulting in misleading and incorrect information (Liu and Welch, 2012). Therefore, it is important that the radiolabeling strategy, radionuclide type, and nanoparticle material are compatible, suited to the study purpose, and possess high *in vivo* stability (Liu and Welch, 2012; Sun et al., 2015). Although exogenous chelation of radionuclides is relatively easy, efficient and low cost, the resulting stability of radiolabels can be potentially problematic. Radionuclides may detach from chelators through trans-chelation or chelators may interact *in vivo* and subsequently disassociate from the nanoparticle (Bass et al., 2000; Boswell et al., 2004; Sun et al., 2015). The attachment of a chelator may also influence or damage the surface properties of nanoparticles, as high temperatures are required for chelation (Lu et al., 2018). This can adversely affect the conjugation capacity of targeting ligands (e.g., antibodies and PEG density), thereby resulting in impaired targeting, reduced circulation times and decreased imaging activity (Chang et al., 2008; Moghimi et al., 2012; Lu et al., 2018). Chelation issues can be avoided with direct bombardment radiolabeling, however this technique is limited by high costs, complexity of use, and the potential to damage the nanoparticles with ion-beam/neutron irradiation (Gibson et al., 2011). While intrinsic radioactive nanoparticles can exhibit high stability with limited radiolabel detachment, potential long-term toxicity and its limited applicability to only a few radioisotope-nanoparticle combinations present challenges for this technique (Liu T. et al., 2012; Chen et al., 2013; Goel et al., 2014). Furthermore, the chelator-free post-synthetic radiolabeling approach is fast, specific and can produce a high labeling yield, however is again limited to only a few nanoparticle and isotope combinations (Chen et al., 2013; Sun et al., 2015).

In comparison to other imaging modalities, PET and SPECT have the advantages of being able to image biochemical processes and are highly sensitive (nanomolar to picomolar level) (Table 1). Therefore, signals can be detected with very small amounts of labels which minimizes the disruption of cell function and surrounding tissue (Townsend, 2004; Ziegler, 2005; Pimlott and Sutherland, 2011; Van Audenhaege et al., 2015). These nuclear medicine imaging techniques are also quantitative and not restricted by tissue penetration limits (Koo et al., 2006; Janib et al., 2010; Kim et al., 2017). Several limitations should be considered for PET and SPECT imaging, including exposure to ionizing radiation, high costs, slow image acquisition times, and inability to be used for longitudinal studies due to radiolabel decay (Kim et al., 2017). Both imaging techniques also have low spatial resolution and provide a lack of anatomic information, hence they are often combined with other imaging modalities such as MRI or CT (Janib et al., 2010; Kim et al., 2017). When comparing between the two imaging techniques, SPECT has low photon detection efficiency and relatively low resolution due to the use of collimation, whereas PET is much more sensitive and provides more radiation event localization data owing to the detection of emissions “coincident” in time (Koo et al., 2006). The positron-emitting isotopes used in PET are also able to be substituted for naturally occurring atoms, thereby enhancing the ability to image molecular events (Massoud and Gambhir, 2003). However, SPECT can image multiple radionuclide probes simultaneously and is more widely available. SPECT scans are also significantly less expensive than PET scans, partly because their radionuclides are simple to prepare, more easily obtained, and typically possess longer half-lives than PET radionuclides (Massoud and Gambhir, 2003; Janib et al., 2010; Pimlott and Sutherland, 2011). In addition, PET typically requires a cyclotron or generator (Massoud and Gambhir, 2003).

## Conclusion

There is a range of techniques available for evaluating the biodistribution of nanoparticles *in vivo*. In general, the choice of technique depends on the: (i) physicochemical characteristics of the nanoparticle formulation; (ii) compatibility and stability of the nanoparticles with different labels and labeling methods; (iii) study duration (single or multiple time points); (iv) analysis type (quantitative or qualitative); (v) sample type (whole animal, whole organ/tissue, or tissue sections); and (vi) degree of detail required (organ/tissue accumulation or cellular association). Other aspects that should be considered include accessibility, costs, accuracy, image resolution, toxicity, complexity, and duration of the procedure. Each technique has its own advantages and limitations, as well as capabilities for assessing real-time, whole-organ, and cellular accumulation. The techniques which allow real-time and qualitative imaging of biodistribution in live animals are *in vivo* optical imaging, CT, MRI and nuclear medicine imaging (PET and SPECT). PET and SPECT are also able to provide quantitative data of uptake into specific organs or tissues, along with LSC and indirectly measuring drug concentration. Of the techniques available, only *in vivo* optimal imaging, CT and MRI are capable of imaging nanoparticle biodistribution across multiple time-points in longitudinal studies. In addition, histology and electron microscopy are the only techniques that can provide detailed information on the cellular association of nanoparticles following *in vivo* administration. Research on the use of other modalities for studying the biodistribution of nanoparticles *in vivo* are currently being explored, including ultrasound imaging of nanoparticles loaded with ultrasound contrast agents (e.g., insoluble gas perfluorocarbons or sulfur hexafluoride) (Janib et al., 2010; Shapiro et al., 2014; Kim et al., 2017). Nanoparticles with multifunctional theranostic capabilities, incorporating multi-mode contrast agents are also rapidly gaining popularity for biomedical applications.

## Author contributions

SH: conception of the work. LA and SH: drafting of the manuscript. SH: preparation of the figure and table. SH, DS, JF, WP, AM, and AW: reviewing the article critically for important intellectual content.

### Conflict of interest statement

The authors declare that the research was conducted in the absence of any commercial or financial relationships that could be construed as a potential conflict of interest.
